# PFAS-Contaminated Pesticides Applied near Public Supply Wells Disproportionately Impact Communities of Color in California

**DOI:** 10.1021/acsestwater.3c00845

**Published:** 2024-05-14

**Authors:** Arianna Libenson, Seigi Karasaki, Lara J. Cushing, Tien Tran, Jenny L. Rempel, Rachel Morello-Frosch, Clare E. Pace

**Affiliations:** † Environmental Science, Policy, and Management, University of California Berkeley, Berkeley, California 94720, United States; ‡ Energy and Resources Group, University of California Berkeley, Berkeley, California 94720 United States; § Fielding School of Public Health, University of California Los Angeles, Los Angeles, California 90095, United States; ∥ Community Water Center, Sacramento and Visalia, California 93291, United States; ⊥ School of Public Health, University of California Berkeley, Berkeley, California 94720, United States

**Keywords:** Environmental Justice, Human Right to Water, Community Water Systems (CWSs), Pollution, Disparities, PFAS, Pesticides

## Abstract

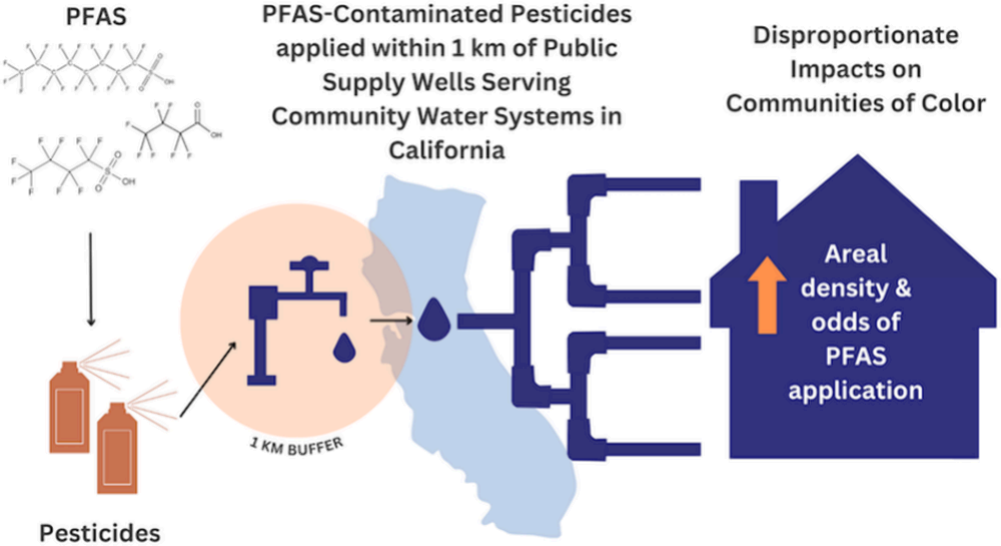

Contaminated drinking water from widespread environmental pollutants such as perfluoroalkyl and polyfluoroalkyl substances (PFAS) poses a rising threat to public health. PFAS monitoring in groundwater is limited and fails to consider pesticides found to contain PFAS as a potential contamination source. Given previous findings on the disproportionate exposure of communities of Color to both pesticides and PFAS, we investigated disparities in PFAS-contaminated pesticide applications in California based on community-level sociodemographic characteristics. We utilized statewide pesticide application data from the California Department of Pesticide Regulation and recently reported concentrations of PFAS chemicals detected in eight pesticide products to calculate the areal density of PFAS applied within 1 km of individual community water systems’ (CWSs) supply wells. Spatial regression analyses suggest that statewide, CWSs that serve a greater proportion of Latinx and non-Latinx People of Color residents experience a greater areal density of PFAS applied and greater likelihood of PFAS application near their public supply wells. These results highlight agroecosystems as potentially important sources of PFAS in drinking water and identify areas that may be at risk of PFAS contamination and warrant additional PFAS monitoring and remediation.

## Abstract 2

Community water systems in California threatened by PFAS contamination from pesticide application serve a higher percent of Latinx and Non-Latinx People of Color populations.

## 1 Introduction

Per- and polyfluoroalkyl substances (PFAS) are a class of synthetic chemicals used ubiquitously in commercial products and industrial processes.^1,2^ Recognized PFAS sources include chrome plating facilities, airports permitted to use PFAS-containing aqueous film-forming foam (AFFF), and military training sites.^3^ PFAS released from point sources through effluent discharge and other media undergo environmental transport through air, soil, surface water, and groundwater interactions, resulting in aquifer contamination.^4−6^ Due to the increasing threat of PFAS to drinking water quality, its ubiquitous detection in human blood samples,^7^ and growing evidence of health effects,^8−11^ several PFAS species are currently undergoing regulatory action under the Safe Drinking Water Act, and the EPA recently announced a final National Primary Drinking Water Regulation for six PFAS.^12^

Current efforts to understand and model PFAS distribution may be limited by missing and incomplete data on sources of PFAS contamination, especially within rural communities. A clustering method used to attribute mixtures of PFAS to different sources in surface water in New York and Rhode Island performed well overall but performed poorly in rural areas where the model failed to attribute PFAS detected in rural surface water to a PFAS source with the same level of reliability as models performed in urban areas.^13^ It is possible that significant PFAS sources may be missing from data sets used in source attribution models, which could explain poor model performance in rural areas. Another study that used machine learning to predict 35 PFAS species in California groundwater performed better for some PFAS species than others but did not include variables for region, rurality, or agricultural area,^14^ so it is unclear if model performance varied by land use type.

Pesticide products have recently come under scrutiny as a potential environmental source of PFAS following the detection of PFAS chemicals (perfluorobutanesulfonic acid [PFBS], perfluorobutanoic acid [PFBA], perfluorooctanesulfonic acid [PFOS], perfluoroheptanesulfonic acid [PFHpS], perfluorooctanoic acid [PFOA], and hexafluoropropylene oxide dimer acid [HFPO–DA]) in varying concentrations in multiple pesticides.^15−17^ Despite ongoing debate about the underlying source of PFAS in pesticides,^18^ this is a significant concern given the breadth and mass of pesticide applications in agricultural areas and our current understanding of water quality challenges already faced by rural, agricultural communities reliant on groundwater.

Eighty-five percent of residents in California rely on groundwater for some or all of their drinking water supply,^19^ typically delivered by community water systems (CWSs; i.e. systems that serve at least 25 people year-round or have at least 15 service connections).^20,21^ In 2022, 376 CWSs serving over 1.2 million Californians were out of compliance with water quality regulations at one point throughout the year.^22^ Small CWSs (i.e., systems with 15–199 service connections) face additional challenges meeting safety standards from regulated drinking water contaminants (i.e., arsenic and nitrate), due to widespread groundwater contamination, aging infrastructure, and a lack of technical and financial resources to meet regulatory standards.^21,23−25^

There is a paucity of data on PFAS in California’s drinking water. The US Environmental Protection Agency’s (EPA) third round of sampling for the Unregulated Contaminant Monitoring Rule (UCMR3) tested for 6 PFAS at the point of delivery (i.e., treated water) between 2013 and 2015 in 456 public drinking water systems^26,27^ and UCMR5 will test for 29 PFAS in an expanded list of water systems.^27,28^ Approximately 24% of national UCMR5 results have been released so far, and testing is expected to conclude in 2025.^27^ The California State Water Resources Control Board (SWRCB) has also released data on 18 PFAS sampled in 2019 and 2020 for 2,915 public supply wells, which represents approximately 12% of municipal supply wells in the state.^56,36^

Although environmental justice research on PFAS is limited, preliminary evidence from Liddie et al. that relied on monitoring data from 18 states (including UCMR3 data and SWRCB sampling in California) revealed that CWSs serving higher proportions of Latinx and non-Latinx Black residents across the U.S. are associated with a greater likelihood of PFAS contamination.^29^ Given previous environmental justice research in California showing that lack of access to safe drinking water–particularly among populations served by small CWSs–disproportionately impacts rural, low-income Latinx communities,^21,23,24^ additional research is needed to elucidate possible inequities in PFAS exposure, especially as new sources of PFAS are discovered. This research can also support regulatory efforts: The California SWRCB has made a formal commitment to support environmental and racial justice, stating that these goals will be achieved when race is no longer a predictor of water quality and all racial and ethnic groups receive equal protection from environmental hazards.^30^

We assessed the threat of PFAS applied near drinking water supplies due to the application of pesticide products. Focusing on the distributive dimension of environmental justice,^31^ we evaluated the spatial applications of eight pesticide products recently found to contain PFAS.^15−17^ We spatially integrated pesticide use data from the California Department of Pesticide Regulation’s (DPR) Pesticide Use Reporting (PUR) program,^32^ sociodemographic data from the US Census Bureau’s American Community Survey (ACS),^33−35^ and public drinking water supply well locations^36^ and CWS service area boundaries^37^ from the SWRCB. We developed statewide estimates of PFAS-pesticide application to calculate the applied areal density of PFAS chemicals.

We evaluated differences in PFAS application by community level racial/ethnic composition; however, we wish to acknowledge the limitation of using racial/ethnic composition as a variable. Race is a social construct, and racial labels reflect and reinforce structural inequities.^38,39^ In the present study, we seek to document whether racial differences exist in PFAS application, investigate what other socially driven factors might contribute, and consider racism (not biological race) as a plausible explanation for observed differences in exposure given that racism alters one’s experiences across the life course in terms of where one lives and their opportunities for education and occupation.^38^

**Figure 1:**
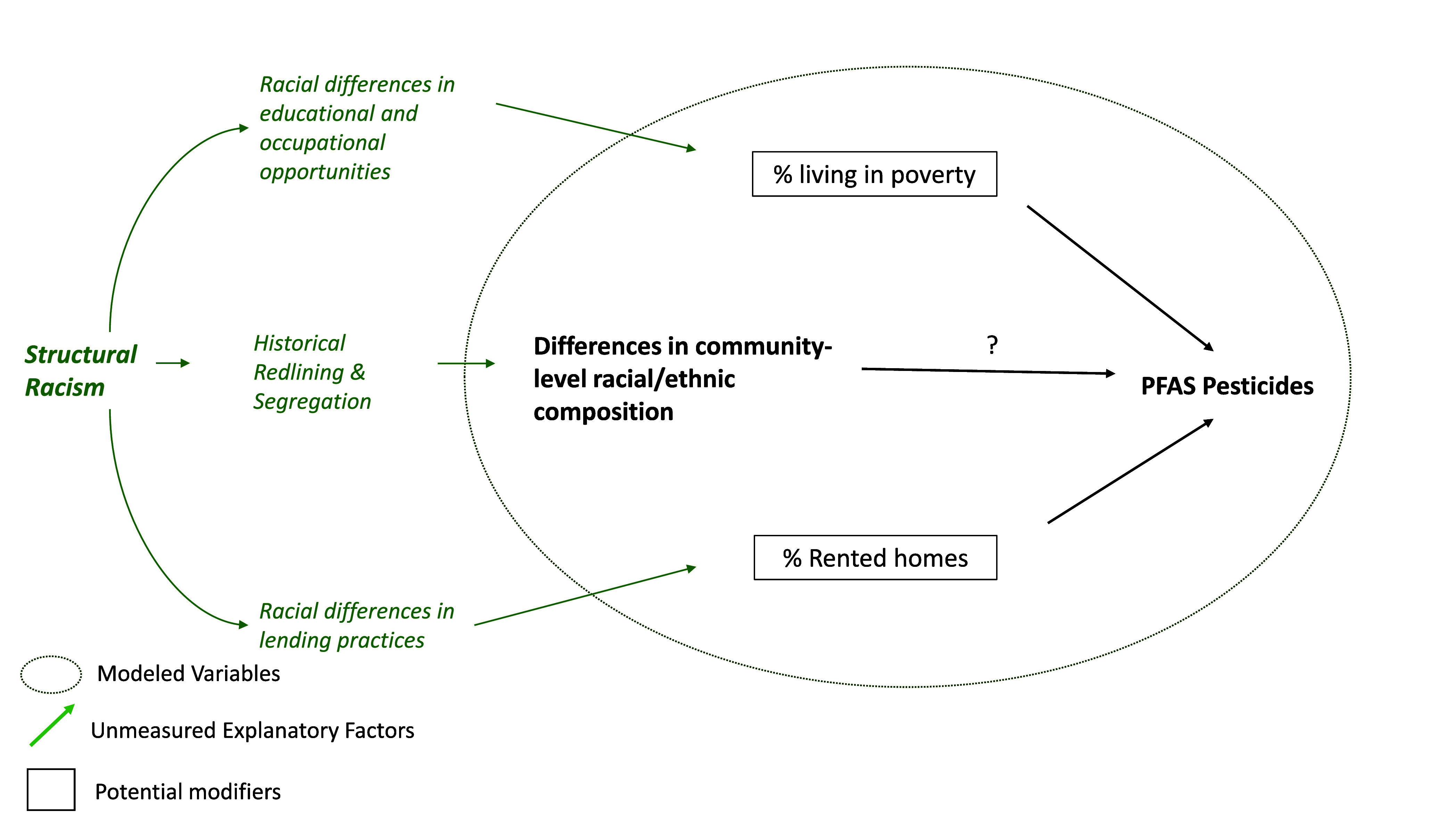
Conceptual Diagram: Relationship between community level racial/ethnic composition and PFAS-pesticides, modified by poverty and housing tenure.

Due to challenges measuring structural racism, we developed a conceptual model to depict the underlying mechanisms and proposed relationships with modeled variables (Figure 1). Racist lending practices have contributed to lower rates of home ownership in communities of Color that persist to the present day.^40^ Disinvestment in education, infrastructure, and a lack of high paying jobs in communities of Color in the US reinforce systems that reduce economic opportunity, limit earning potential, and have resulted in higher rates of poverty.^38^ In the present study, we evaluated population characteristics across two estimates of PFAS burden, and we tested the hypothesis that poverty and housing tenure modify observed inequities in PFAS application experienced by communities of Color.

## 2 Data and Methods

We conducted a statewide analysis to evaluate the threat of groundwater contamination from PFAS in pesticides to public supply wells serving CWSs in California. We applied spatial methods to estimate CWS demographics, CWS supply well locations, and total PFAS (i.e., sum of calculated PFAS mass from each pesticide product) applied between 2019 and 2021. We then used this data set to evaluate associations between PFAS estimates and sociodemographic characteristics of populations served by CWSs.

### 2.1 Community Water System Boundaries

We used CWS service area boundaries provided by the SWRCB and Cal EPA’s Office of Environmental Health Hazard Assessment (OEHHA) representing “active” water systems according to California’s Safe Drinking Water Information System (SDWIS), as of 2020.^37^ OEHHA processed boundaries by removing duplicates and assigning overlapping areas to the smaller water system. As an additional processing step, we manually fixed boundaries where system consolidations were confirmed but not yet reflected in state-maintained boundaries (Figure S1).^37^

### 2.2 Public Supply Well Locations

We obtained public supply well locations for CWSs from the Groundwater Ambient Monitoring and Assessment (GAMA) tool.^36^ We generated 1 km buffers around each public supply well to approximate their impact area and dissolved buffered areas by water system ID. The selection of a 1 km buffer was informed by methods developed by OEHHA to define the average distance at which polluted sites pose a threat to nearby groundwater quality for the Groundwater Threats layer of CalEnviroScreen 4.0.^41^

### 2.3 Community Water System Population Characteristics

CWS population was estimated using a tiered approach integrating multiple data sources: 1) high-resolution (100 m^2^) gridded population estimates;^42^ 2) State government data on water system population and service connection counts;^43^ and 3) a point-location data set of statewide domestic well reliance within water system boundaries.^44^ Although the SWRCB maintains records of water system service population, we derived our own estimates because the sum of state estimates exceeded the total population of California.

First, we summed the residential population within each water system boundary according to high-resolution gridded population estimates.^42^ If the summed population for a given water system was under 25 (i.e., below the technical definition of a CWS), we substituted SDWIS population estimates (*n* = 434).^43^ For a small number of these systems (*n* = 10) for which SDWIS population data was unavailable, we estimated their service populations based on their number of service connections (assuming each service connection serves an average of 3 people).^45^ We further adjusted for the possibility of reliance on domestic wells within CWS boundaries using a high-resolution data set of domestic wells joined to residential parcels and addresses to estimate the number of people reliant on domestic wells.^44^ We subtracted this domestic well population from the CWS service area population and used these refined estimates of the CWS population to calculate descriptive statistics.

We characterized sociodemographic variables at the CWS level, using data from the ACS 5-year estimates from 2016 to 2020 at the scale of census block groups; we included variables for racial/ethnic composition,^34^ household tenure,^33^ and poverty.^35^ Racial/ethnic identity–a self-identified classification from the ACS–was considered because of racial inequities in federal infrastructure investment and protections from environmental hazards.^46^ We calculated the proportion of rented households; the population that is Hispanic/Latinx, non-Latinx (NL) White, NL Black, NL Asian, NL Native American, NL Other/two or more, and NL People of Color (POC) (which includes NL Black, NL Asian, NL Native American, and NL Other/two or more); and households living in poverty (defined as a household income under twice the federal poverty level). We referred to Hispanic/Latinx populations as “Latinx” due to the preference of our community partners, as well as the fact that the majority of California’s Hispanic residents are of Latin American heritage.^47^

We assigned block-group level sociodemographic variables to individual CWS boundaries using areal apportionment.^37^ This was necessary because of spatial differences between water systems and block groups: a single water system may serve more than one block group, and portions of the same block group are frequently served by different water systems. Finally, we calculated regional and statewide descriptive statistics for water system sociodemographic characteristics. Regions were defined by previous studies (Bay Area, Central Coast, Eastern Sierra, Inland Empire/Imperial Desert, Northern California, Northern Sierra, San Joaquin Valley, Southern California (Figure S2).^21,24,48^

### 2.4 Calculated Applied PFAS from Pesticides

We used the California DPR’s PUR database to calculate the statewide application of PFAS from pesticides.^32^ The DPR’s PUR program requires monthly reporting of all legal agricultural pesticide applications within the state including application to greenways, cemeteries, rangeland, pastures, along the roadside, and railroad rights-of-way. We identified 12 individual pesticide brands representing eight pesticide products with evidence of PFAS contamination (Table S1).^15−17^ We summed the total pounds (lbs.) of the PFAS-pesticides by the Public Land Survey System (PLSS) section (i.e., approximately one square mile), by year, and across the 3-year study period (2019–2021). Each PLSS section received a value representing the cumulative sum across 2019 through 2021, selected to encompass the most recent years of available data and to align with reports of PFAS detections in pesticide products.^15−17^ Next, we calculated the amount of individual PFAS (perfluorobutanesulfonic acid [PFBS], perfluorobutanoic acid [PFBA], and perfluorooctanesulfonic acid [PFOS]) and total PFAS (combination of PFBS, PFBA, and PFOS) applied in milligrams (mg) within each PLSS section by year and between 2019 and 2021 using eq 1 (PFHpS, PFOA, and HFPO–DA were not applied in California during the study period)
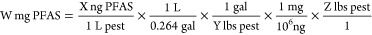
1where W is the mass of an individual PFAS chemical applied in mg; X is the original PFAS concentration reported in nanograms (ng) per L of PFAS-pesticide; Y is the product-specific density of the PFAS-pesticide in lbs per gallon; and Z is the sum of PFAS-pesticides applied in lbs within each PLSS section between 2019 and 2021.

**Figure 2:**
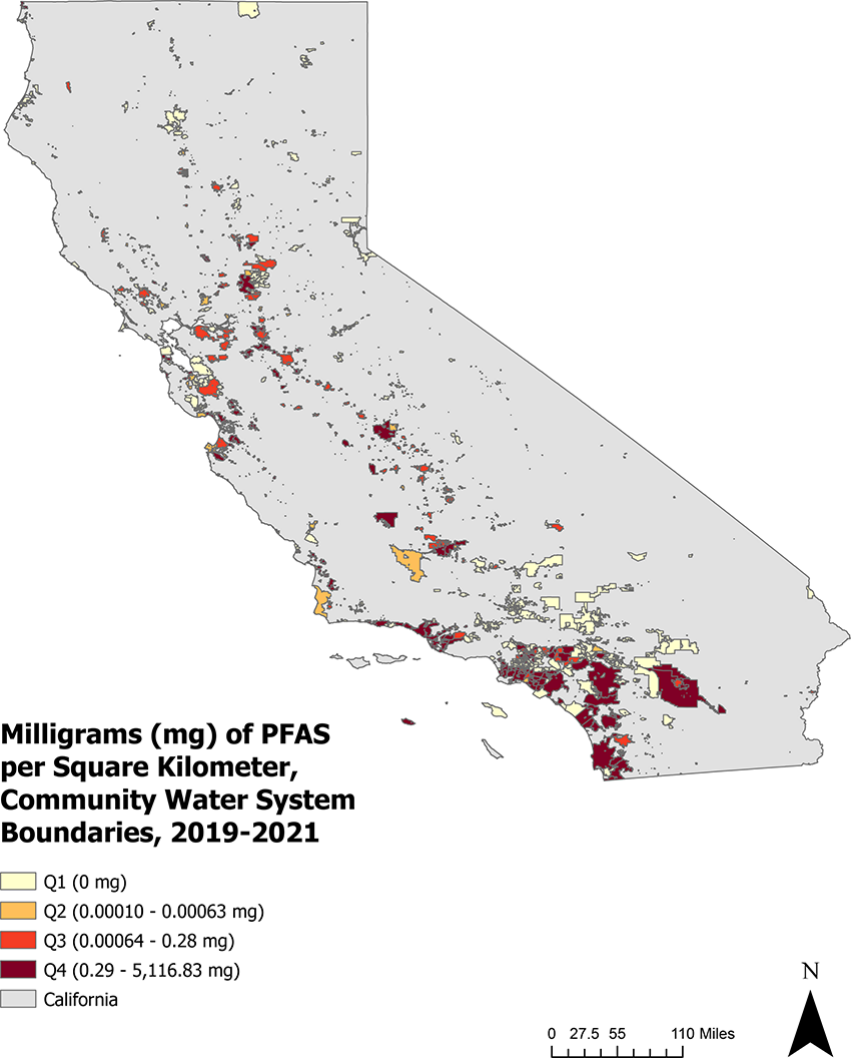
Areal density (milligrams per square kilometer) of PFAS (combination of PFBS, PFBA, and PFOS) applied between 2019 to 2021 within 1 km of public supply wells serving community water systems (CWSs) in California. PFAS application is displayed as quartiles within CWS service area boundaries.

Next, we assigned PFAS to the buffer areas. We intersected PLSS layers containing individual and total PFAS with buffered public supply well areas and calculated the areal density of PFAS (mg/km^2^) applied within 1 km of public supply wells serving each CWS (Figure 2). We then calculated regional and statewide descriptive statistics for the PFAS.

### 2.6 Statistical Analysis

We used two-part generalized additive models (GAMs) to estimate the associations between PFAS (mg/km^2^) with selected sociodemographic variables across individual CWS observations (*n* = 732), and binary outcomes representing the application of any PFAS (*n* = 2,444). Two-part models, commonly used to model discrete-continuous outcomes, allowed for handling the zeros (*n* = 1,712) and positive values (*n* = 732) separately.^49^ We scaled continuous predictor variables by 10%, including racial/ethnic composition (% Latinx, % non-Latinx [NL] People of Color [POC], and % NL White [reference = NL White]), housing tenure (% rented), and % poverty. We fit penalized cubic regression splines for population density (people/100 m^2^) and for latitude and longitude of the centroid of service area boundary (decimal degrees) to account for CWS size and spatial autocorrelation, respectively.

For continuous data, we used generalized additive linear regression models presented in the Supporting Information (eq S1). The outcome variable was the log-transformed milligrams of PFAS applied per square km. We specified a Gaussian error distribution and identity link to estimate geometric mean ratios.

We used generalized additive logistic regression models (eq S2) to evaluate binary outcomes for the application of any PFAS within 1 km of water system supply wells. A binomial distribution and logit link function were specified for logistic models to estimate odds ratios. We adjusted for the number of public supply wells in the logistic models to account for the buffer area size.

We examined residual spatial autocorrelation using Moran’s I test statistic and inspection of model residuals. We assessed the model fit using the Akaike information criterion (AIC), log-likelihood, and diagnostic plots.

We conducted sensitivity analyses for both the linear and logistic regression analysis in which PFOS was excluded and PFBA+PFBS were retained. This was done because the large contribution of PFOS to the total PFAS-chemical application may have obscured the contribution of other PFAS.

### 2.7 Software

We conducted data processing in R version 4.1.2 (R Foundation, Vienna, Austria) and ArcGIS version 3.0.3 (ESRI, Redlands, CA). We performed statistical analyses in R version 4.1.2 (R Foundation, Vienna, Austria) using the following packages: rgdal,^50^ spdep,^51^ and mgcv.^52^

## 3 Results and Discussion

### 3.1 Regional Characteristics of Community Water Systems

We estimated that 28.4 million Californians are served by 2,444 active CWSs that rely fully or partially on groundwater (i.e., the water system is associated with at least one public supply well) (Table 1). A large percentage of these systems (24.0%) were in the San Joaquin Valley (SJV)–the primary agricultural region of California–and served approximately 3.7 million SJV residents. Residents served by groundwater-reliant CWSs in the SJV represented 13.0% of the state’s population, and they experienced a disproportionate burden of poverty (38.4%) compared to the statewide average (27.0%). Nearly half (49.4%) of the population served by water systems in the SJV self-identified as Latinx, compared to the statewide average of 36.5%.

**Table 1:** Sociodemographic Characteristics and PFAS-Contaminated Pesticide Use across Community Water Systems (CWSs) in California (CA), by Region^a^

Region (*n* = CWSs)^b^	Bay Area (*n* = 281)	Central Coast (*n* = 346)	Eastern Sierra (*n* = 138)	Inland Empire/Imperial (*n* = 236)	Northern CA (*n* = 378)	Northern Sierra (*n* = 150)	San Joaquin Valley (*n* = 586)	Southern CA (*n* = 329)	Statewide (*n* = 2444)
Population Served	4,747,071	1,365,341	64,981	4,414,922	605,280	2,075,754	3,705,481	11,430,777	28,409,608
All PFAS^c^ (mg/km^2^)	2.7	41.5	2.4 × 10^–06^	12.8	1.4	1.8	3.9	18.7	11.6
PFBS^c^ (mg/km^2^)	2.4 × 10^–04^	7.3 × 10^–04^	2.4 × 10^–06^	3.8 × 10^–04^	4.9 × 10^–04^	4.8 × 10^–04^	5.1 × 10^–03^	7.9 × 10^–04^	1.6 × 10^–03^
PFBA^c^ (mg/km^2^)	6.9 × 10^–05^	1.6 × 10^–03^	0.0	9.3 × 10^–04^	2.0 × 10^–05^	4.1 × 10^–05^	1.2 × 10^–04^	8.3 × 10^–04^	5.2 × 10^–04^
PFOS^c^ (mg/km^2^)	2.7	41.5	0.0	12.8	1.4	1.8	3.9	18.7	11.6
% non-Latinx White^d^	38.7	49.7	75.8	35.3	72.8	49.9	34.1	34.9	38.2
% Latinx^d^	20.5	39.1	16.7	47.9	16.8	22.1	49.4	37.9	36.5
% non-Latinx Black^d^	4.1	1.4	0.5	5.8	1.5	6.7	4.1	5.2	4.8
% non-Latinx Asian^d^	31.5	4.4	2.0	7.0	3.2	15.3	8.8	16.0	15.3
% non-Latinx Native American^d^	0.2	0.3	1.8	0.4	1.6	0.3	0.4	0.8	0.6
% non-Latinx Other^d^	4.5	3.2	3.2	3.1	4.1	5.2	3.1	3.4	3.6
% living in poverty^d^	16.2	24.7	27.4	31.3	35.6	30.2	38.4	25.3	27.0
Mean % rented^e^	30.5	30.8	26.8	30.6	27.3	34.1	32.3	34.6	30.9

a Socioeconomic variables were accessed from the US Census Bureau’s American Community Survey (ACS) 2020 5-year estimates and assigned to water system service boundaries using areal apportionment.

b Our universe of Community Water Systems (CWSs) is limited to those served by public supply wells.

c All PFAS refers to the sum of milligrams of PFBS, PFBA, and PFOS applied per km^2^ within public supply well buffer areas via PFAS-pesticide application between 2019 and 2021.

d Value represents mean across water systems in region. Denominator is the regional population served by CWCs.

e Mean % rented reflects household-level estimates.

### 3.2 Regional and Statewide PFAS Application

Statewide, 732 water systems were within 1 km of PFAS application, serving an estimated 18.5 million Californians, which corresponds to over 65% of the of the population included in the present study and about 22% of the state’s total population in 2020. We estimated that a combined total of 229,978 mg of PFOS, PFBS, and PFBA were applied within 1 km of public supply wells via contaminated pesticide products between 2019 to 2021 (Table S2). The vast majority (99.9% or 229,932.8 mg) was PFOS. In contrast, PFBS and PFBA accounted for 32.7 and 10.4 mg, respectively (Table S2). The Central Coast, Southern California, and Inland Empire/Imperial Desert had the highest areal density of total PFAS applied (Table 1). The SJV had the highest areal density of PFBS applied, followed by Southern California and the Central Coast. PFBA was applied most heavily in the Central Coast, Inland Empire/Imperial Desert, and Southern California (Table 1).

### 3.3 Population Characteristics and PFAS Application

We evaluated population characteristics at the community water system scale across two indicators of PFAS burden: the areal density of PFAS and odds of PFAS application. In unadjusted models, a higher proportion of % Latinx and % NL POC population was associated with increased areal density of PFAS and increased likelihood that PFAS were applied (Tables S4, S5). We progressively adjusted our models for spatial factors and potential effect modifiers (% poverty and % rented) (Tables S6, S7). Our results for higher proportion Latinx were robust and remained statistically significant in progressively and fully adjusted models. In fully adjusted models, a 10% higher proportion in the Latinx population was associated with a 27% increase in the areal density of PFAS in mg/km^2^ (GMR = 1.27; 95% CI = 1.05, 1.54) and a 60% increased odds of PFAS application (OR = 1.60; 95% CI = 1.48, 1.74) (Table 2). In contrast, a higher proportion of NL POC was significant in unadjusted models and did not retain statistical significance in progressively or fully adjusted models, despite only minor changes in effect estimates (Tables S6, S7).

**Table 2:** Two-Part Generalized Additive Model Results Estimating the Association between Sociodemographic Variables and PFAS Application among Community Water Systems, California, 2019-2021^a^

**Independent Variables**	**Geometric Mean Ratios**^b^**Milligrams of PFAS**^d^**per km**^**2**^**(*n*^e^ = 732**)	**Odds Ratios**^c^**PFAS applied (Yes/No)(*n*^e^ = 2,444)**
% Latinx	1.27 (1.05–1.54)	1.60 (1.48–1.74)
% non-Latinx People of Color	1.34 (0.99–1.82)	1.11 (0.99–1.24)
% poverty	0.79 (0.60–1.04)	1.00 (0.90–1.10)
% rented	1.17 (0.94–1.44)	1.07 (0.98–1.16)
Number of public supply wells	NA^f^	1.07 (1.05–1.08)
AIC	4,202.67	1,967.72
Log likelihood	–2,078.55	–844.31
Moran’s I P-value	0.88	0.77

a Socioeconomic variables were accessed from the U.S. Census Bureau’s American Community Survey (ACS) 2016–2020 5-year estimates and assigned to water system service boundaries using areal apportionment. Models included fitted splines for population density (people/100 m^2^) and for latitude and longitude (decimal degrees).

b Geometric mean ratio assessing PFAS application with respect to sociodemographic characteristics.

c Odds ratio assessing likelihood of PFAS application with respect to sociodemographic characteristics.

d PFAS refers to the sum of milligrams of PFBS, PFBA, and PFOS applied per km^2^ within public supply well buffer areas via PFAS-pesticide application.

e *n* refers to the number of community water systems included in each model.

f Number of supply wells was excluded because the outcome variable was already adjusted for buffer area, a variable related to the number of supply wells.

The lack of statistical significance for NL POC in the adjusted models may have resulted from data limitations. We were unable to evaluate models that further characterized people of color into self-identified categories (i.e., NL Black, NL Native American, etc.) due to low representation of each racial/ethnic group in CWS populations. Although necessary for this analysis, our approach of grouping racial/ethnic categories may have obscured racial inequities in the PFAS threat experienced by subpopulations. Our results highlight the complexity of disentangling relationships between community-level racial/ethnic composition and measures of social vulnerability and suggest that more granular data may be necessary to fully understand the factors involved.

It is worth noting that a higher proportion of poverty and a higher proportion of rented households were both associated with the increased odds of PFAS-pesticides in unadjusted bivariate models (Table S5), but this relationship was not significant in the main analysis (Table 2) or sensitivity analysis (Table S3), suggesting that poverty and housing tenure are not the primary drivers of observed relationships, although additional studies are needed to more fully explain the complex relationship between racial/ethnic composition and socioeconomic factors. Despite challenges modeling the relationship between structural racism and environmental injustice, our interpretation of the relationship between racial/ethnic composition and socioeconomic factors align with our hypothesis that structural racism plays a critical role in exposures that differ by measured race/ethnicity. We believe that the modeled variables in this study (i.e., % rented homes and % poverty) modify the relationship between community-level racial/ethnic composition and exposure to PFAS-pesticides given the modeled variables influence on a community’s ability to address distributive aspects of environmental injustice, which can lead to heightened environmental exposures.^53^

We conducted a sensitivity analysis in which PFOS was excluded and PFBA+PFBS was retained that revealed similar results to our main analysis: a 10% higher proportion in Latinx population was positive and significantly associated with the areal density and odds of PFBA+PFBS application; % NL POC was not significant in either model (Table S3).

Our results support tracking the SWRCB’s environmental justice goals^30^ and demonstrate that racial/ethnic composition is significantly associated with PFAS application within 1 km of public supply wells in models that account for housing tenure, poverty, number of supply wells, population density, and spatial autocorrelation. These findings echo concerns raised by a recent nationwide study that looked more generally at the spatial risk of PFAS to CWSs and found that CWS watersheds with PFAS sources serve higher proportions of Latinx and NL Black residents compared to those without a PFAS source.^29^ Although it remains to be determined by future research, it is possible that PFAS in community water supplies, originating from nearby PFAS-pesticide applications, may contribute to existing patterns in the uneven distribution of PFAS sources^29^ and to an ongoing legacy of disproportionate impacts from environmental risks (including pesticides) impacting communities of color.

Our results are consistent with previous studies in California that found disproportionate levels of pesticide application related to community-level sociodemographic characteristics on various spatial scales. In a statewide analysis, Cushing et al. found that, compared to areas with predominantly NL White residents, areas with larger populations of Latinx and POC (including African American, Native American, Asian/Pacific Islander, and or multiracial individuals) were associated with higher use of hazardous and volatile pesticides.^48^ In Ventura County, Temkin et al. also found that toxic pesticides were disproportionately applied in communities with higher percentages of Latinx and POC populations.^54^ It is worth noting that these studies evaluated pesticide use in specific locations but did not consider PFAS-containing pesticide products specifically; however, we believe that similar underlying mechanisms, such as structural racism, are likely responsible.

A previous statewide study that also evaluated associations between housing tenure and agricultural-related contamination had similar results to ours; Pace et al. did not detect an association between renter status and nitrate concentrations in water from CWSs or domestic wells.^21^ In contrast, a regional study of CWSs conducted by Balazs et al. identified increased nitrate contamination in drinking water served to communities in the SJV with higher percentages of rented households compared to communities with higher percentages of home ownership.^23^ Better measures of housing vulnerability may be needed to understand statewide associations between socioeconomic characteristics and water quality or PFAS threat.

It is worth noting that studies have reported inconsistent findings regarding the presence and range of PFAS concentrations in pesticides. Lasee et al. reported levels of PFOS several orders of magnitude higher than studies conducted by the Center for Biological Diversity (CBD) and PEER.^15−17^ A verification analysis conducted by the EPA failed to detect measurable levels of PFOS or other PFAS species tested for by Lasee et al. in the same pesticide products, possibly due to differences in sample preparation methods.^55^ Results from PEER (2020) were confirmed through independent testing done by the Massachusetts Department of Environmental Protection and the EPA;^16^ results from CBD/PEER (2023) have yet to be verified.^17^ Inconsistencies in PFAS concentrations in pesticides may be due to incomplete information on the extent of contamination; only a small fraction of pesticide products have been tested for a limited suite of PFAS chemicals by a limited number of studies. Our results should be interpreted carefully with the understanding that we are estimating the potential for groundwater contamination by PFAS based on the application of PFAS-pesticides near public supply wells; however, we did not directly measure actual concentrations of PFAS in public supply wells or in groundwater.

We leveraged publicly available secondary data from several sources, each with its own inherent limitations. For example, rural areas with lower populations are represented by larger block groups, and population characteristics averaged across rural or urban block groups do not capture heterogeneity in the community characteristics. CWS boundaries may be inaccurate due to system expansions and consolidations. In order to estimate potential PFAS threats to public supply wells, we made several assumptions, including that pesticide products contained consistent concentrations of PFAS over the study period and that all PFAS contamination was accounted for. Additionally, our use of a 1 km buffer area for public supply wells is smaller than used in other studies, which use watersheds.^29^ As a result, we may have underestimated potential threats from PFAS-pesticide applications. Overall, this study underscores the pressing need for additional research on PFAS contamination in pesticides and groundwater.

## 4 Conclusions

We found significant statewide disparities in the threat of groundwater contamination by PFAS in pesticides applied near community water supply wells, indicating a distributive environmental injustice from this newly regulated environmental health hazard. As more research and data become available, future studies should explore the relationship between applications of PFAS-contaminated pesticides and measured concentrations of PFAS in drinking water from nearby CWSs. Due to data limitations, we likely underestimated PFAS applications. Nevertheless, our results indicate racial and ethnic disparities in potential PFAS threats to CWS, thus raising environmental justice concerns.

Despite our use of racial/ethnic composition as a model variable, we wish to emphasize that significant differences between racialized groups do not account for biological or cultural differences. Our inability to measure and model structural racism is a limitation, and future studies will benefit from evaluating more nuanced outcomes, such as intersectional relationships between race and socioeconomic variables that were beyond the scope of this study. Moving forward, it will also be imperative to consider the 1.6 million people served by domestic wells, which likely face even greater contamination threats due to the fact that their water quality is unregulated by state and federal authorities.^44^ Future research should include other sources of agriculture-related PFAS, such as the use of contaminated biosolids from wastewater treatment facilities as agricultural fertilizer. This study informs monitoring and remediation, promotes enhanced PFAS testing in rural areas, highlights environmental justice concerns, and supports State efforts to achieve racial equity and the Human Right to Water in California.

## Supporting Information Available

Additional details on PFAS-contaminated pesticide products and sensitivity analysis results: Table S1. PFAS-Contaminated Pesticide Product Details; Table S2. PFAS Application Statewide and Within Public Supply Well Buffer Areas (1 km), California; Table S3. Two-Part Generalized Additive Model Results: Sensitivity Analysis Estimating the Association Between Sociodemographic Variables and PFBA+PFBS Application, 2019–2021, Among Community Water Systems, California; Table S4. Geometric Mean Ratios for Unadjusted Bivariate Log–Linear Models Estimating the Association Between Sociodemographic Variables and PFAS Application Among Community Water Systems, California, 2019–2021; Table S5. Odds Ratios for Unadjusted Bivariate Logistic Models Estimating the Association Between Sociodemographic Variables and PFAS Application Among Community Water Systems, California, 2019–2021; Table S6. Geometric Mean Ratios for Progressively Adjusted Log–Linear Models Estimating the Association Between Sociodemographic Variables and PFAS Applications Among Community Water Systems, California, 2019–2021; Table S7. Odds Ratios for Progressively Adjusted Logistic Models Estimating the Association Between Sociodemographic Variables and PFAS Application Among Community Water Systems, California, 2019–2021; Figure S1. Flowchart of modifications made to state-maintained water system boundaries; Figure S2. Regions in California; Equation S1. Linear model to estimate associations between sociodemographics and PFAS application; Equation S2. Logistic model to estimate associations between sociodemographics and PFAS application (PDF)

## References

[ref1] Sunderland E. M.; Hu X. C.; Dassuncao C.; Tokranov A. K.; Wagner C. C.; Allen J. G. A review of the pathways of human exposure to poly- and perfluoroalkyl substances (PFASs) and present understanding of health effects. J. Expo Sci. Environ. Epidemiol 2019, 29 (2), 131–147. 10.1038/s41370-018-0094-1. 30470793 PMC6380916

[ref2] US Environmental Protection Agency. EPA’s Per- and Polyfluoroalkyl Substances (PFAS) Action Plan. 2019. Accessed May 2, 2024. https://www.epa.gov/sites/default/files/2019-02/documents/pfas_action_plan_021319_508compliant_1.pdf.

[ref3] Hu X. C.; Andrews D. Q.; Lindstrom A. B.; et al. Detection of Poly- and Perfluoroalkyl Substances (PFASs) in U.S. Drinking Water Linked to Industrial Sites, Military Fire Training Areas, and Wastewater Treatment Plants. Environ. Sci. Technol. Lett. 2016, 3 (10), 344–350. 10.1021/acs.estlett.6b00260. 27752509 PMC5062567

[ref4] Weber A. K.; Barber L. B.; LeBlanc D. R.; Sunderland E. M.; Vecitis C. D. Geochemical and Hydrologic Factors Controlling Subsurface Transport of Poly- and Perfluoroalkyl Substances, Cape Cod, Massachusetts. Environ. Sci. Technol. 2017, 51 (8), 4269–4279. 10.1021/acs.est.6b05573. 28285525

[ref5] Guelfo J. L.; Korzeniowski S.; Mills M. A.; et al. Environmental Sources, Chemistry, Fate, and Transport of Per- and Polyfluoroalkyl Substances: State of the Science, Key Knowledge Gaps, and Recommendations Presented at the August 2019 SETAC Focus Topic Meeting. Environ. Toxicol. Chem. 2021, 40 (12), 3234–3260. 10.1002/etc.5182. 34325493 PMC8745034

[ref6] Interstate Technology and Regulatory Council. Fate and Transport of Per- and Polyfluoroalkyl Substances (PFAS). Published online July 2022. Accessed May 2, 2024. https://pfas-1.itrcweb.org/wp-content/uploads/2022/09/FT_PFAS_Fact_Sheet_083122_508.pdf.

[ref7] Centers for Disease Control and Prevention. National Report on Human Exposure to Environmental Chemicals. Published online September 28, 2023. 10.15620/cdc:105345.

[ref8] Agency for Toxic Substances and Disease Registry. Potential health effects of PFAS chemicals. Published November 1, 2022. Accessed October 12, 2023. https://www.atsdr.cdc.gov/pfas/health-effects/index.html.

[ref9] Barry V.; Winquist A.; Steenland K. Perfluorooctanoic Acid (PFOA) Exposures and Incident Cancers among Adults Living Near a Chemical Plant. Environ. Health Perspect. 2013, 121 (11–12), 1313–1318. 10.1289/ehp.1306615. 24007715 PMC3855514

[ref10] Grandjean P.; Budtz-Jørgensen E. Immunotoxicity of perfluorinated alkylates: calculation of benchmark doses based on serum concentrations in children. Environ. Health. 2013, 12 (1), 35 10.1186/1476-069X-12-35. 23597293 PMC3643874

[ref11] Liu G.; Dhana K.; Furtado J. D.; et al. Perfluoroalkyl substances and changes in body weight and resting metabolic rate in response to weight-loss diets: A prospective study. PLOS Med. 2018, 15 (2), e1002502 10.1371/journal.pmed.1002502. 29438414 PMC5810983

[ref12] US Environmental Protection Agency. Per- and Polyfluoroalkyl Substances (PFAS). Published April 10, 2024. Accessed April 27, 2024. https://www.epa.gov/sdwa/and-polyfluoroalkyl-substances-pfas.

[ref13] Zhang X.; Lohmann R.; Dassuncao C.; et al. Source attribution of poly- and perfluoroalkyl substances (PFASs) in surface waters from Rhode Island and the New York Metropolitan Area. Environ. Sci. Technol. Lett. 2016, 3 (9), 316–321. 10.1021/acs.estlett.6b00255. 28217711 PMC5310642

[ref14] Dong J.; Tsai G.; Olivares C. I. Prediction of 35 Target Per- and Polyfluoroalkyl Substances (PFASs) in California Groundwater Using Multilabel Semisupervised Machine Learning. ACS EST Water. 2024, 4, 969 10.1021/acsestwater.3c00134.

[ref15] Lasee S.; McDermett K.; Kumar N.; et al. Targeted analysis and Total Oxidizable Precursor assay of several insecticides for PFAS. J. Hazard Mater. Lett. 2022, 3, 100067 10.1016/j.hazl.2022.100067.

[ref16] Public Employees for Environmental Responsibility (PEER). Aerially Sprayed Pesticide Contains PFAS. Published online December 1, 2020. Accessed August 11, 2023. https://peer.org/aerially-sprayed-pesticide-contains-pfas/.

[ref17] Public Employees for Environmental Responsibility (PEER). High Levels of Dangerous ‘Forever Chemicals’ Found in California’s Most-Used Insecticide. Published online May 2, 2023. Accessed August 11, 2023. https://peer.org/dangerous-forever-chemicals-n-californias-insecticide/.

[ref18] US Environmental Protection Agency. Per- and Polyfluoroalkyl Substances (PFAS) in Pesticide and Other Packaging. Published February 15, 2024. Accessed March 10, 2024. https://www.epa.gov/pesticides/pfas-packaging.

[ref19] Hanak E.; Chappelle C.; Harter T. Groundwater in California. Public Policy Institute of California. Published May 2017. Accessed November 16, 2023. https://www.ppic.org/publication/groundwater-in-california/.

[ref20] Chappelle C.; Collins J.; Hanak E. Access to Safe Drinking Water in California. Public Policy Institute of California. Accessed October 24, 2023. https://www.ppic.org/publication/access-to-safe-drinking-water/.

[ref21] Pace C.; Balazs C.; Bangia K.; et al. Inequities in Drinking Water Quality Among Domestic Well Communities and Community Water Systems, California, 2011–2019. Am. J. Public Health 2022, 112 (1), 88–97. 10.2105/AJPH.2021.306561. 34936392 PMC8713636

[ref22] Abhold K.; Allen W.; Altevogt A. 2023 Drinking Water Needs Assessment. California State Water Resources Control Board; 2023: p 21. Accessed May 2, 2024.https://www.waterboards.ca.gov/drinking_water/certlic/drinkingwater/documents/needs/2023needsassessment.pdf.

[ref23] Balazs C.; Morello -Frosch R.; Hubbard A.; Ray I. Social Disparities in Nitrate-Contaminated Drinking Water in California’s San Joaquin Valley. Environ. Health Perspect 2011, 119 (9), 1272–1278. 10.1289/ehp.1002878. 21642046 PMC3230390

[ref24] Bangia K.; August L.; Slocombe A.; Faust J. Assessment of contaminants in California drinking water by region and system size. AWWA Water Sci. 2020, 2 (5), e1194 10.1002/aws2.1194.

[ref25] Balazs C. L.; Ray I. The Drinking Water Disparities Framework: On the Origins and Persistence of Inequities in Exposure. Am. J. Public Health. 2014, 104 (4), 603–611. 10.2105/AJPH.2013.301664. 24524500 PMC4025716

[ref26] US Environmental Protection Agency. Third Unregulated Contaminant Monitoring Rule. Published September 1, 2015. Accessed October 9, 2023. https://www.epa.gov/dwucmr/third-unregulated-contaminant-monitoring-rule.

[ref27] US Environmental Protection Agency. Occurrence Data from the Unregulated Contaminant Monitoring Rule. Published September 1, 2015. Accessed November 29, 2023. https://www.epa.gov/dwucmr/occurrence-data-unregulated-contaminant-monitoring-rule.

[ref28] US Environmental Protection Agency. Fifth Unregulated Contaminant Monitoring Rule. Published January 11, 2021. Accessed November 17, 2023. https://www.epa.gov/dwucmr/fifth-unregulated-contaminant-monitoring-rule.

[ref56] GeoTracker PFAS Map data. https://geotracker.waterboards.ca.gov/map/pfas_map. Accessed May 2022.

[ref36] California State Water Resources Control Board. Municipal Wells Dataset, Groundwater Ambient Monitoring and Assessment (GAMA), Groundwater Information System. Published online 2023. Accessed November 17, 2023. https://gamagroundwater.waterboards.ca.gov/gama/gamamap/public/.

[ref29] Liddie J. M.; Schaider L. A.; Sunderland E. M. Sociodemographic Factors Are Associated with the Abundance of PFAS Sources and Detection in U.S. Community Water Systems. Environ. Sci. Technol. 2023, 57 (21), 7902–7912. 10.1021/acs.est.2c07255. 37184106 PMC10233791

[ref30] California State Water Resources Control Board. Environmental Justice. Published March 17, 2023. Accessed March 9, 2024. https://www.waterboards.ca.gov/water_issues/programs/outreach/education/justice.html.

[ref31] Holifield R. DEFINING ENVIRONMENTAL JUSTICE AND ENVIRONMENTAL RACISM. Urban Geogr. 2001, 22 (1), 78–90. 10.2747/0272-3638.22.1.78.

[ref32] California Department of Pesticide Regulation. Pesticide Use Reporting. Accessed February 7, 2023. https://www.cdpr.ca.gov/docs/pur/purmain.htm.

[ref33] U.S. Census Bureau. B25003: TENURE - Census Bureau Table. 2016–2020 American Community Survey 5-Year Estimates. Accessed March 9, 2023. https://data.census.gov/table?q=Table+B25003&g=0400000US06$1500000&tid=ACSDT5Y2020.B25003.

[ref34] U.S. Census Bureau. B03002: HISPANIC OR LATINO ORIGIN··· - Census Bureau Table. 2016–2020 American Community Survey 5-Year Estimates. Accessed March 9, 2023. https://data.census.gov/table?q=Table+B03002&g=0400000US06$1500000&tid=ACSDT5Y2020.B03002.

[ref35] U.S. Census Bureau. C17002: RATIO OF INCOME TO POVERTY··· - Census Bureau Table. 2016–2020 American Community Survey 5-Year Estimates. Accessed March 9, 2023. https://data.census.gov/table?q=Table+C17002&g=0400000US06$1500000&tid=ACSDT5Y2020.C17002.

[ref37] Pace C.; Fisher E.; Subramanian A.; Cushing L.; Morello-Frosch R. Water System Boundaries Version 2.0. Published online 2023. Accessed May 2, 2024. https://drinkingwatertool.communitywatercenter.org/wp-content/uploads/2023/09/CWS_v2_082123_Metadata_final-1.pdf.

[ref38] Payne-Sturges D. C.; Gee G. C.; Cory-Slechta D. A. Confronting Racism in Environmental Health Sciences: Moving the Science Forward for Eliminating Racial Inequities. Environ. Health Perspect. 2021, 129 (5), 055002 10.1289/EHP8186. 33945300 PMC8096378

[ref39] Roberts D. Fatal Invention: How Science, Politics, and Big Business Re-Create Race in the Twenty-First Century. New Press/ORIM; 2011.

[ref40] Berberian A. G.; Rempel J.; Depsky N.; Bangia K.; Wang S.; Cushing L. J. Race, Racism, and Drinking Water Contamination Risk From Oil and Gas Wells in Los Angeles County, 2020. Am. J. Public Health 2023, 113 (11), 1191–1200. 10.2105/AJPH.2023.307374. 37651660 PMC10568503

[ref41] August L.; Bangia K.; Plummer L.; CalEnviroScreen 4.0. California Office of Environmental Health Hazard Assessment; 2021: pp 113–120. Accessed May 2, 2024. https://oehha.ca.gov/media/downloads/calenviroscreen/report/calenviroscreen40reportf2021.pdf.

[ref42] Depsky N. J.; Cushing L.; Morello-Frosch R. High-resolution gridded estimates of population sociodemographics from the 2020 census in California. PLoS One 2022, 17 (7), e0270746 10.1371/journal.pone.0270746. 35834564 PMC9282657

[ref43] US Environmental Protection Agency. Safe Drinking Water Information System (SDWIS) Federal Reporting Services. Published November 23, 2015. Accessed November 17, 2023. https://www.epa.gov/ground-water-and-drinking-water/safe-drinking-water-information-system-sdwis-federal-reporting.

[ref44] Rempel J.; Pace C.; Cushing L.; Morello-Frosch R. Domestic Well Areas Version 2.0. Published online 2023. Accessed May 2, 2024. https://drinkingwatertool.communitywatercenter.org/wp-content/uploads/2023/09/DWA_v2_plss_083123_Metadata.pdf.

[ref45] California State Water Resources Control Board. Determination of Population Served by a Public Water System. Accessed November 17, 2023. https://drinc.ca.gov/ear/Methodology.htm.

[ref46] Braveman P. A.; Cubbin C.; Egerter S.; Williams D. R.; Pamuk E. Socioeconomic Disparities in Health in the United States: What the Patterns Tell Us. Am. J. Public Health 2010, 100 (S1), S186–S196. 10.2105/AJPH.2009.166082. 20147693 PMC2837459

[ref47] McGhee E. California’s Hispanic Community. Public Policy Institute of California. Published October 5, 2022. Accessed December 7, 2023. https://www.ppic.org/blog/californias-hispanic-community/.

[ref48] Cushing L.; Faust J.; August L. M.; Cendak R.; Wieland W.; Alexeeff G. Racial/Ethnic Disparities in Cumulative Environmental Health Impacts in California: Evidence From a Statewide Environmental Justice Screening Tool (CalEnviroScreen 1.1). Am. J. Public Health 2015, 105 (11), 2341–2348. 10.2105/AJPH.2015.302643. 26378826 PMC4605180

[ref49] Belotti F.; Deb P.; Manning W. G.; Norton E. C. Twopm: Two-Part Models. Stata J. 2015, 15 (1), 3–20. 10.1177/1536867X1501500102.

[ref50] Bivand R. S.; Rundel C. rgdal: Bindings for the “Geospatial” Data Abstraction Library. Published online 2021. Accessed May 2, 2024. https://CRAN.R-project.org/package=rgdal.

[ref51] Bivand R.; Piras G. Comparing Implementations of Estimation Methods for Spatial Econometrics. J. Stat Softw. 2015, 63, 1–36. 10.18637/jss.v063.i18.

[ref52] Wood S. N. Generalized Additive Models: An Introduction with R, 2nd ed. Chapman & Hall/CRC Press; 2017.

[ref53] Adkins-Jackson P. B.; Chantarat T.; Bailey Z. D.; Ponce N. A. Measuring Structural Racism: A Guide for Epidemiologists and Other Health Researchers. Am. J. Epidemiol. 2022, 191 (4), 539–547. 10.1093/aje/kwab239. 34564723 PMC9077112

[ref54] Temkin A. M.; Uche U. I.; Evans S.; et al. Racial and social disparities in Ventura County, California related to agricultural pesticide applications and toxicity. Sci. Total Environ. 2022, 853, 158399 10.1016/j.scitotenv.2022.158399. 36063919

[ref55] Qian Y.; French D. Verification Analysis for PFAS in Pesticide Products (ACB Project B23–05b). Published online May 18, 2023. Accessed May 2, 2024. https://www.epa.gov/system/files/documents/2023-05/BEAD%20PFAS%20Study%20Results%202023.pdf.

