# Supplementary material for: PFAS-Contaminated Pesticides Applied near Public Supply Wells Disproportionately Impact Communities of Color in California
Source: ACS ES T Water. 2024 May 14;4(6):2495–503. doi: 10.1021/acsestwater.3c00845 (PMC11186009; doi:10.1021/acsestwater.3c00845)
Supplement: Supplementary file 1 — ew3c00845_si_001.pdf [file ew3c00845_si_001.pdf]

PFAS-Contaminated Pesticides Applied near Public Supply Wells Disproportionately Impact Communities of Color in California

Arianna Libenson,[1] Seigi Karasaki,[2] Lara J. Cushing[3], Tien Tran,[4] Jenny L. Rempel,[2] Rachel Morello-Frosch,[*1,5] Clare E. Pace[*1]

1. University of California Berkeley, Environmental Science, Policy, and Management, Berkeley, California, United States
2. University of California Berkeley, Energy and Resources Group, Berkeley, California, United States
3. University of California Los Angeles, Fielding School of Public Health, Los Angeles, California, United States
4. Community Water Center, Sacramento and Visalia, California, United States
5. University of California Berkeley, School of Public Health, Berkeley, California United States

Supporting Information

Table S1. PFAS-Contaminated Pesticide Product Details

Pesticide Product	Manufacturer	CA DPR Registration Numbers[a]	Current DPR Status	DPR Date Active[b]	PFAS Concentration Detected	Study[c]
AVID 0.15 EC	Syngenta	100-896-ZD<br>100-896-AA<br>100-896-ZA	Active<br>Inactive<br>Inactive	1998	3.9± 0.5 ppm PFOS	Lasee et al., 2022
Pedestal	Chemtura	66222-40-AA-400<br>66222-40-ZA-400	Inactive<br>Inactive	2002	9.2± 0.3 ppm PFOS	Lasee et al., 2022

Ultra-Pure Oil	BASF	69526-5-ZA-499<br>69526-5-AA-499	Active<br>Inactive	2007	8.6 ±0.7 ppm PFOS	Lasee et al., 2022
Marathon 1%	OHP	59807-15-AA	Active	2015	13.3± 1.4 ppm PFOS	Lasee et al., 2022
Oberon 2SC	Bayer	264-719-AA	Active	2005	19.2± 1.2 ppm PFOS,<br>1,500 ppt PFBA	Lasee et al., 2022;<br>PEER, 2023
Malathion 5EC	Drexel	19713-217-AA	Active	2007	17.8± 0.7 ppm PFOS,<br>510 ppt PFOA,<br>680 ppt PFHpS	Lasee et al., 2022;<br>PEER, 2023
Intrepid 2F	Dow AgroSciences	62719-442-AA	Active	2003	350 ppt PFBS	PEER, 2023
Anvil 10 + 10	Clarke Mosquito Control Products, Inc.	1021-1688-AA-8329	Active	2000	250 ppt PFOA,<br>260 – 500 ppt HFPO-DA,	PEER, 2020

Note:

[a] Registration numbers were accessed from the California Department of Pesticide Regulation Product/Label Database portal. Multiple brands were listed if we could not identify the specific brand tested in a study.

[b] If multiple product brands were identified, the active date listed is the earliest registration of any brand.

[c] Study that reported measurable levels of PFAS in the pesticide product(s).[1-3]

Table S2. PFAS Application Statewide and within Public Supply Well Buffer Areas (1km), California

	All PFAS[a] (mg)	PFOS[a] (mg)	PFBS[a] (mg)	PFBA[a] (mg)	PFHpS[a,b]	PFOA[a,b]
Statewide	1,600,408	1,599,623	688.5	48.7	27.3	20.5
Within Public Supply Buffer Area (%)[c]	229,978 (14.4%)	229,932.8 (14.4%)	32.7 (4.8%)	10.4 (21.3%)	0 (0%)	0 (0%)

Note:

[a] PFAS application from 2019-2021 is reported in milligrams (mg).

[b] Although perfluorooctanoic acid (PFOA) and perfluoroheptanesulfonic acid (PFHpS) were detected in Malathion 5EC, this product was not found to have been applied within the public supply well buffer areas likely due to its presence on the DPR's Groundwater Protection List which restricts the use of specific pesticides near drinking water wells.[4]

[c] Percentages were calculated by dividing the total PFAS applied in buffer areas by statewide total.

Table S3. Two-Part Generalized Additive Model Results: Sensitivity Analysis Estimating the Association Between Sociodemographic Variables and PFBA+PFBS Application Among Community Water Systems, California, 2019-2021

**Independent Variables**	**Geometric Mean Ratios[a] Milligrams of PFBA+PFBS[c] per km^2 (n[d]=639)**	**Odds Ratios[b] PFBA+PFBS[c] applied (Yes/No) (n[d]=2,444)**
% Latinx	1.28 (1.14 - 1.45)	1.61 (1.48 - 1.75)
% non-Latinx People of Color	1.10 (0.89 - 1.35)	1.03 (0.90 - 1.16)
% poverty	0.84 (0.71 - 1.00)	1.01 (0.91 - 1.12)
% rented	1.13 (0.98 - 1.29)	1.08 (0.99 - 1.17)
Number of public supply wells	NA[e]	1.05 (1.03 - 1.06)
AIC	2,968.92	1,782.65
Log likelihood	-1445.35	-774.56
Moran's I P-value	0.96	0.94

Note. Socioeconomic variables were accessed from the U.S. Census Bureau's American Community Survey (ACS) 2016-2020 5-year estimates and assigned to water system service

boundaries using areal apportionment. Models included fitted splines for population density (people/100 square meters) and for latitude and longitude (decimal degrees).

[a] Geometric mean ratio assessing PFBA+PFBS application with respect to sociodemographic characteristics.

[b] Odds ratio assessing likelihood of PFBA+PFBS application with respect to sociodemographic characteristics.

[c] The sum of milligrams of PFBA and PFBS applied per km2 within public supply well buffer areas via pesticide application.

[d] n refers to the number of Community water systems included in each model.

[e] Number of supply wells excluded because outcome already adjusted for buffer area, a variable related to the number of supply wells.

Table S4. Geometric Mean Ratios for Unadjusted Bivariate Log-Linear Models Estimating the Association Between Sociodemographic Variables and PFAS Application Among Community Water Systems, California, 2019-2021

Geometric Mean Ratios[a] Milligrams of PFAS[b] per km^2 (n[c]=732)				
**Independent Variables**	% Latinx[d]	% non-Latinx People of Color[d]	% Poverty	% Rented
**Unadjusted estimate**	1.20 (1.02 – 1.41)	2.48 (1.79 – 3.46)	0.54 (0.44-0.67)	0.98 (0.80-1.20)
**AIC**	4,529.10		4,522.80	4,555.90
**Log likelihood**	-2,260.56		-2,258.40	-2,274.94
**Moran's I P-value**	<2.2e-16		<2.2e-16	<2.2e-16

Note. Socioeconomic variables were accessed from the U.S. Census Bureau's American Community Survey (ACS) 2016-2020 5-year estimates and assigned to water system service boundaries using areal apportionment.

[a] Geometric mean ratio assessing PFAS application with respect to sociodemographic characteristics.

[b] PFAS refers to the sum of milligrams of PFBS, PFBA, and PFOS applied per km^2 within public supply well buffer areas via PFAS-pesticide application.

[c] n refers to the number of community water systems included in each model.

[d] %Latinx and %non-Latinx People of Color [Reference Group: % non-Latinx White] were included as racial/ethnic categories in the same unadjusted bivariate model.

Table S5. Odds Ratios for Unadjusted Bivariate Logistic Models Estimating the Association Between Sociodemographic Variables and PFAS Application Among Community Water Systems, California, 2019-2021

Odds Ratios[a] PFAS[b] applied (Yes/No) (n[c] =2,444)				
**Independent Variables**	% Latinx[d]	% non-Latinx People of Color[d]	% Poverty	% Rented
**Unadjusted estimate**	1.46 (1.40 – 1.52)	1.15 (1.07 – 1.24)	1.11 (1.06-1.17)	1.23 (1.17-1.28)
**AIC**	2,585.70		2,968.90	2,905.40
**Log likelihood**	-1,289.83		-1,482.43	-1450.71
**Moran's I P-value**	<2.2e-16		<2.2e-16	<2.2e-16

Note. Socioeconomic variables were accessed from the U.S. Census Bureau's American Community Survey (ACS) 2016-2020 5-year estimates and assigned to water system service boundaries using areal apportionment.

[a] Odds ratio assessing likelihood of PFAS application with respect to sociodemographic characteristics.

[b] PFAS refers to any amount (or none) of PFBS, PFBA, and PFOS applied within public supply well buffer areas via PFAS-pesticide application.

[c] n refers to the number of community water systems included in each model.

[d] %Latinx and %non-Latinx People of Color [Reference Group: % non-Latinx White] were included as racial/ethnic categories in the same unadjusted bivariate model.

Table S6. Geometric Mean Ratios for Progressively Adjusted Log-Linear Models Estimating the Association Between Sociodemographic Variables and PFAS Application Among Community Water Systems, California, 2019-2021

Geometric Mean Ratios[a] Milligrams of PFAS[b] per km^2 (n[c]=732)			
**Independent Variables**	**+Coordinates[d]**	**+Population Density[e]**	**+Additional Modifier[f] (% Rented)**
% Latinx	1.21 (1.00 - 1.47)	1.29 (1.06 - 1.56)	1.27 (1.05 - 1.54)
% non-Latinx POC	1.24 (0.91 - 1.68)	1.35 (1.00 - 1.84)	1.34 (0.99 - 1.82)
% Poverty	0.80 (0.63 - 1.02)	0.87 (0.68 - 1.11)	0.79 (0.60 - 1.04)
% Rented	NA[g]	NA[g]	1.17 (0.94 - 1.44)
AIC	4,222.68	4,203.15	4,202.67
Log likelihood	-2,091.41	-2,079.96	-2,078.55
Moran's I P-value	0.82	0.87	0.88

Note. Socioeconomic variables were accessed from the U.S. Census Bureau's American Community Survey (ACS) 2016-2020 5-year estimates and assigned to water system service boundaries using areal apportionment. Each column indicates the subsequent addition of an adjustment variable to the model (in addition to the others already adjusted for in the columns to the right).

[a] Geometric mean ratio assessing PFAS application with respect to sociodemographic characteristics.

[b] PFAS refers to the sum of milligrams of PFBS, PFBA, and PFOS applied per km^2 within public supply well buffer areas via PFAS-pesticide application.

[c] n refers to the number of community water systems included in each model.

[d] Addition of coordinates (centroid of water system service area boundaries in decimal degrees) as adjustment variable in the model.

[e] Addition of population density (people/100 square meters) as adjustment variable in the model.

[f] Addition of modifier (% rented households) as adjustment variable in the model.

[g] Models do not yet adjust for % rented households as a modifier.

Table S7. Odds Ratios for Progressively Adjusted Logistic Models Estimating the Association Between Sociodemographic Variables and PFAS Application Among Community Water Systems, California, 2019-2021

Odds Ratios[a] PFAS[b] applied (Yes/No) (n[c] =2,444)				
**Independent Variables**	**+Coordinates[d]**	**+Population Density[e]**	**+Number of Public Supply Wells[f]**	**+Additional Modifier[g] (% Rented)**
% Latinx	1.55 (1.45 - 1.67)	1.58 (1.46 - 1.70)	1.60 (1.48 - 1.72)	1.60 (1.48 - 1.74)
% non-Latinx POC	1.15 (1.03 - 1.27)	1.16 (1.04 - 1.29)	1.12 (1.00 - 1.25)	1.11 (0.99 - 1.24)
% Poverty	0.98 (0.90 - 1.07)	1.02 (0.93 - 1.11)	1.02 (0.93 - 1.12)	1.00 (0.90 - 1.10)
% Rented	NA[h]	NA[h]	NA[h]	1.07 (0.98 - 1.16)
Number of public supply wells	NA[i]	NA[i]	1.07 (1.05 - 1.08)	1.07 (1.05 - 1.08)
AIC	2,132.18	2,062.19	1,984.64	1,967.72
Log likelihood	-951.12	-904.30	-862.70	-844.31
Moran's I P-value	0.89	0.83	0.73	0.7

Note. Socioeconomic variables were accessed from the U.S. Census Bureau's American Community Survey (ACS) 2016-2020 5-year estimates and assigned to water system service boundaries using areal apportionment. Each column indicates the subsequent addition of an adjustment variable to the model (in addition to the others already adjusted for in the columns to the right).

[a] Odds ratio assessing likelihood of PFAS application with respect to sociodemographic characteristics.

[b] PFAS refers to any amount (or none) of PFBS, PFBA, and PFOS applied within public supply well buffer areas via PFAS-pesticide application.

[c] n refers to the number of community water systems included in each model.

[d] Addition of coordinates (centroid of water system service area boundaries in decimal degrees) as adjustment variable in the model.

[e] Addition of population density (people/100 square meters) as adjustment variable in the model.

[f] Addition of the number of public supply wells as adjustment variable in the model.

[g] Addition of modifier (% rented households) as adjustment variable in the model.

[h] Models do not yet adjust for % rented households as a modifier.

[i] Models do not yet adjust for the number of public supply wells.

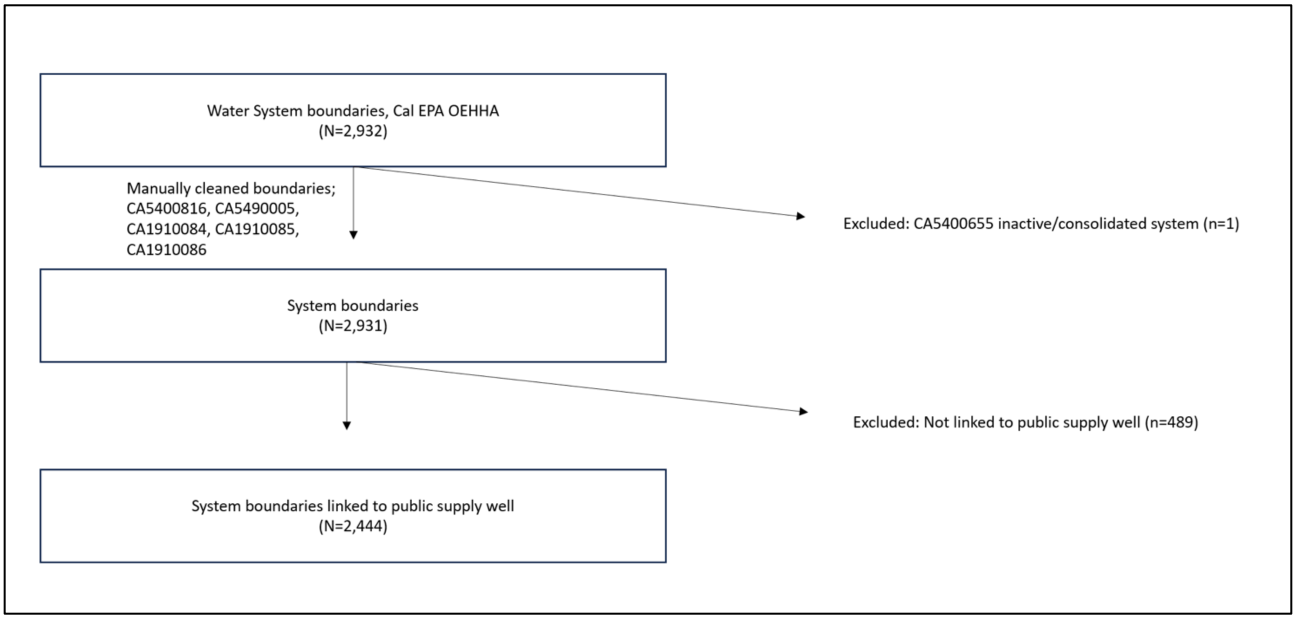

Figure S1. Flow chart of modifications made to state-maintained water system boundaries.

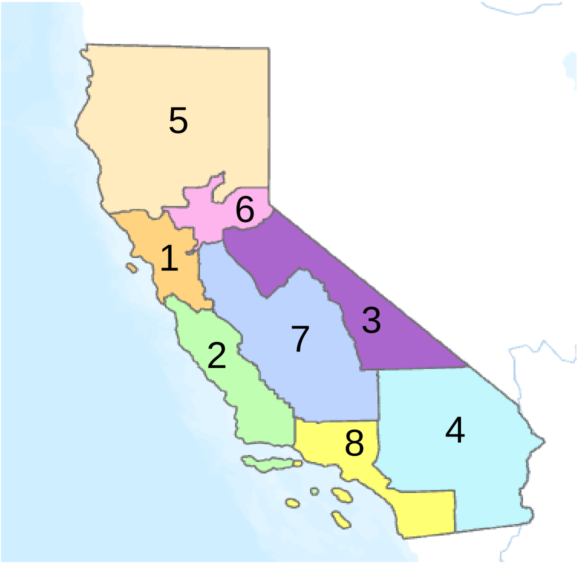

1. Bay Area
2. Central Coast
3. Eastern Sierra
4. Inland Empire/Imperial Desert
5. Northern California
6. Northern Sierra
7. San Joaquin Valley
8. Southern California

Figure S2. Regions in California

Equation S1. Linear model to estimate associations between sociodemographics and PFAS application.

$$\log(areal\ density_i) = \beta_0 + \beta_{1_{\mathrm{Latinx_i}}} + \beta_{2_{\mathrm{NLPOC_i}}} + \beta_{3_{\mathrm{HouseTenure_i}}} + \beta_{4_{\mathrm{Poverty_i}}} + \sum_{i=6}^{9}(\mathrm{s(pop\ density_i)} + \mathrm{s(latitude_i)} + \mathrm{s(longitude_i)}) + \epsilon_i \quad (s1)$$

Equation S2. Logistic model to estimate associations between sociodemographics and PFAS application.

$$\text{Logit}[\Pr(f(applied_i))] = \beta_0 + \beta_{1_{\text{Latinx}_i}} + \beta_{2_{\text{NLPOC}_i}} + \beta_{3_{\text{HouseTenure}_i}} + \beta_{4_{\text{Poverty}_i}} + \beta_{5_{\text{Number of wells}_i}} + \sum_{i=7}^{10} (\, s(\text{pop density}_i) + s(\text{latitude}_i) + s(\text{longitude}_i)) + \epsilon_i$$

(s2)

References

1. Lasee S, McDermett K, Kumar N, et al. Targeted analysis and Total Oxidizable Precursor assay of several insecticides for PFAS. Journal of Hazardous Materials Letters. 2022;3:100067. doi:10.1016/j.hazl.2022.100067

2. Public Employees for Environmental Responsibility (PEER). High Levels of Dangerous 'Forever Chemicals' Found in California's Most-Used Insecticide. Published online May 2, 2023. Accessed August 11, 2023. https://peer.org/dangerous-forever-chemicals-n-californias-insecticide/

3. Public Employees for Environmental Responsibility (PEER). Aerially Sprayed Pesticide Contains PFAS. Published online December 1, 2020. Accessed August 11, 2023. https://peer.org/aerially-sprayed-pesticide-contains-pfas/

4. California Department of Pesticide Regulation. Cal. Code Regs. Tit.3, Section 6800 - Groundwater Protection List. Accessed February 7, 2023. https://www.cdpr.ca.gov/docs/legbills/calcode/040101.htm
